# Subcutaneous anterior pelvic bridge — an innovative technique for fixation of selective acetabular fracture: a case series and literature review

**DOI:** 10.1007/s00264-022-05460-8

**Published:** 2022-06-08

**Authors:** Chien Han Chen, Fang Chieh Lien

**Affiliations:** 1 Present Address: Department of Neurosurgery, Chung Shan Medical University Hospital, 402 Taichung, Taiwan; 2 Department of Orthopedics, Ditmanson Medical Foundation Chia-Yi Christian Hospital, 600 Chiayi, Taiwan; 3 Institute of Medicine, Chung Shan Medical University, Taichung, Taiwan

**Keywords:** Acetabular fracture, Anterior pelvic internal fixation, Pelvic bridge technique, Subcutaneous anterior pelvic bridge, Percutaneous anterior pelvic bridge, Minimally invasive

## Abstract

**Introduction:**

The aim of the study was to introduce an innovative technique involving the use of a subcutaneous anterior pelvic bridge (SAPB) in the treatment of selective acetabular fractures.

**Methods:**

We performed a retrospective study of 21 patients with acetabular fracture who were treated with SAPB between January 2016 and March 2021. The patients’ data were retrieved from electronic charts. Radiological results were evaluated according to the Matta system to assess the quality of the reduction and time of union. Functional outcomes were assessed in line with the d’Aubigné and Postel scoring system. Post-operative complications were also recorded.

**Results:**

SAPB required around 60 minutes, with minimal blood loss and short learning curve. Matta score revealed excellent radiological outcomes in seventeen displaced fractures with seven excellent outcomes and nine good outcomes. Functional outcomes were excellent in twelve hips, good in seven hips, and fair in two hips. Six patients had transient lateral femoral cutaneous nerve palsy.

**Discussion:**

The innovative SAPB method for the treatment of selective acetabular fracture is proven to be a feasible method with promising outcomes. SAPB is a minimally invasive technique and strengthens the stability of fixation, with less blood loss and fewer intra-operative/post-operative complications.

## Introduction

Acetabular fractures are infrequent fractures that are mostly caused by high-energy trauma such as road traffic accidents and sometimes by low-energy trauma such as falling. An anterior column-based fracture pattern is becoming more commonplace than either posterior wall fractures or both-column fractures [1, 2]. A recent study revealed marked changes in the occurrence of fracture types, with a significant rise in anterior column–based fractures (anterior column and associated posterior hemi-transverse), whereas all other fracture patterns have decreased over time [3].

Open reduction and internal fixation (ORIF), coupled with rapid rehabilitation, is regarded as the gold standard for the treatment of acetabular fractures [4]. However, common approaches such as the ilioinguinal and the modified Stoppa approaches require extensive exposure, which could lead to excessive blood loss, infection, neurovascular injuries, and inferior wound healing [5]. An innovative method of fixation for pelvic fracture, which we herein name subcutaneous anterior pelvic bridge (SAPB) [6], has been developed in recent years. SAPB strengthens the stability of pelvic ring fracture. In some specific cases [7], SAPB has been performed independently without posterior pelvic fixation, and the outcome was similar to that of SAPB combined with pelvic fixation [8]. This technique is, however, used mainly for anterior pelvic ring fracture [7]. Little has been reported about the application of SAPB in acetabular fractures. In this retrospective study, we discuss 21 cases of selective acetabular fracture that were treated with SAPB in our hospital and were evaluated for clinical and post-operative characteristics. We also review the literature to better understand the effectiveness of this innovative method for selective acetabular fractures.

### Patients and methods

**Fig. 1:**
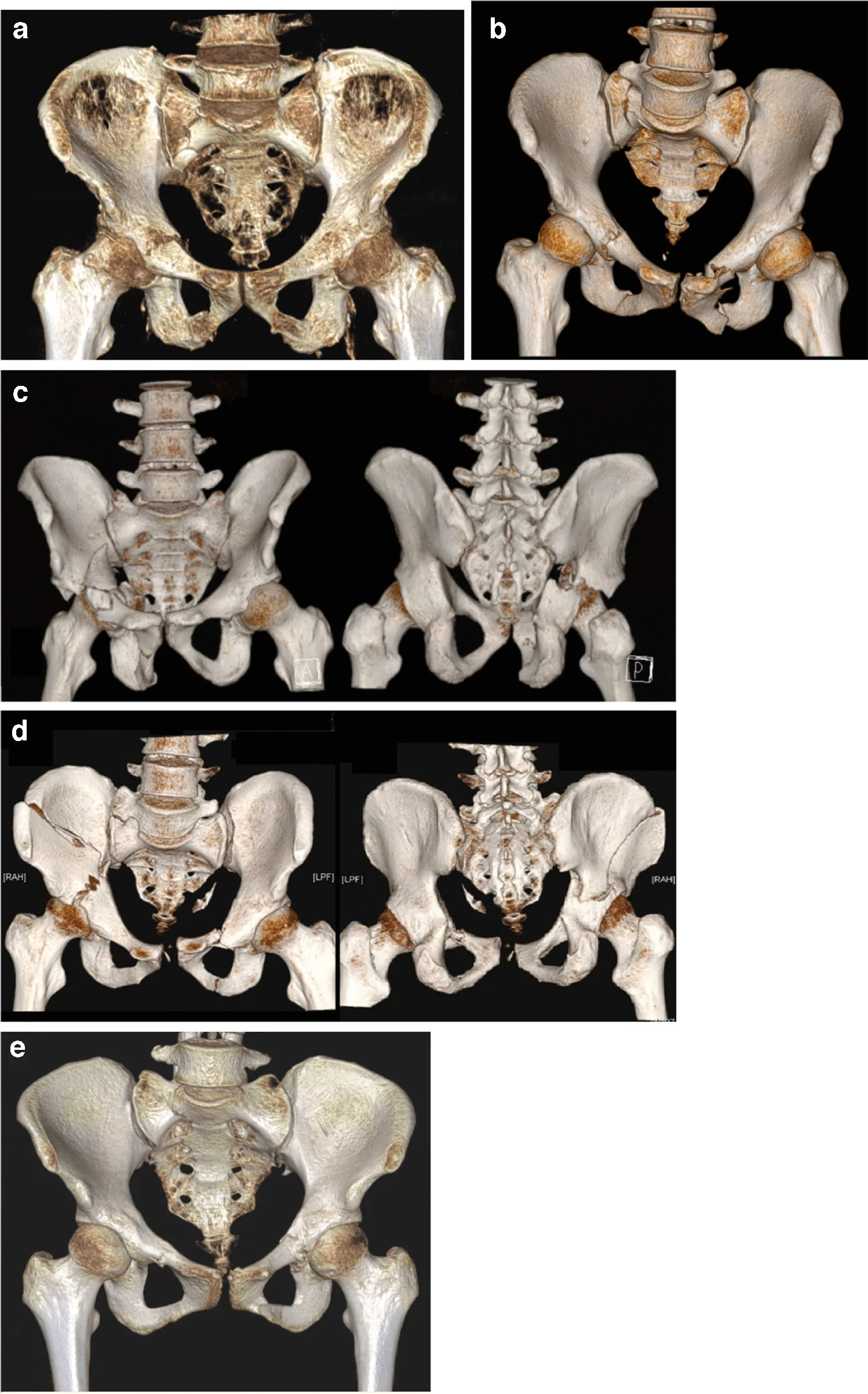
**a** Anterior wall fracture 62A3.1 (no. 16). **b** Left very low anterior column fracture 62A3.3 (no. 17). **c** Transverse fracture 62B3.3 (no. 1). **d** Both column fracture 62C1 (no. 9). **e** Non-displaced acetabular fracture 62A3.1 (no. 20)

This study was approved by our institution’s Ethical Review Board. We conducted a retrospective study to evaluate the short-term outcomes of SAPB in acetabular fracture. All patients underwent closed reduction internal fixation (CRIF) along with SAPB. Our diagnosis was based on pelvic CT scan with 3D reconstruction of the fractures. All cases were confirmed as acetabular fractures and graded by an orthopaedic surgeon according to the AO/OTA classification system. Demographic data and mechanisms of injury were recorded. The surgical criteria for displaced acetabular fractures were a 2-mm or more displacement in the dome area of the acetabulum, the roof arc angle measurement < 45°, and the presence of intra-articular fragments. The surgical criteria for non-displaced acetabular fractures were patients presented with moderate to severe pain after two weeks of conservative treatment and patients presented with other fracture required surgical intervention. Our cases involved the following selective types of acetabular fractures (Fig. 1): (1) anterior wall fracture 62A3.1; (2) very low anterior column fracture 62A3.3; (3) selective transverse fracture 62B2.2, 62B3.3; (4) selective both column fracture 62C1, 62C2e, 62C3, 62C3b, 62C3.1e; (5) multiple injuries with non-displaced acetabular fracture; (6) non-displaced acetabular fracture with persistent pain.

Patients were excluded if the fracture was (1) simple posterior wall/column and (2) anterior column fracture above the iliopubic eminence without posterior column involvement.

Thirteen traumatic patients who received anterior pelvic bridge plate between January 2016 and March 2021 met the criteria for acetabular fractures at our institution. All patients were followed up for a minimum of 12 months.

### Surgical technique

**Fig. 2:**
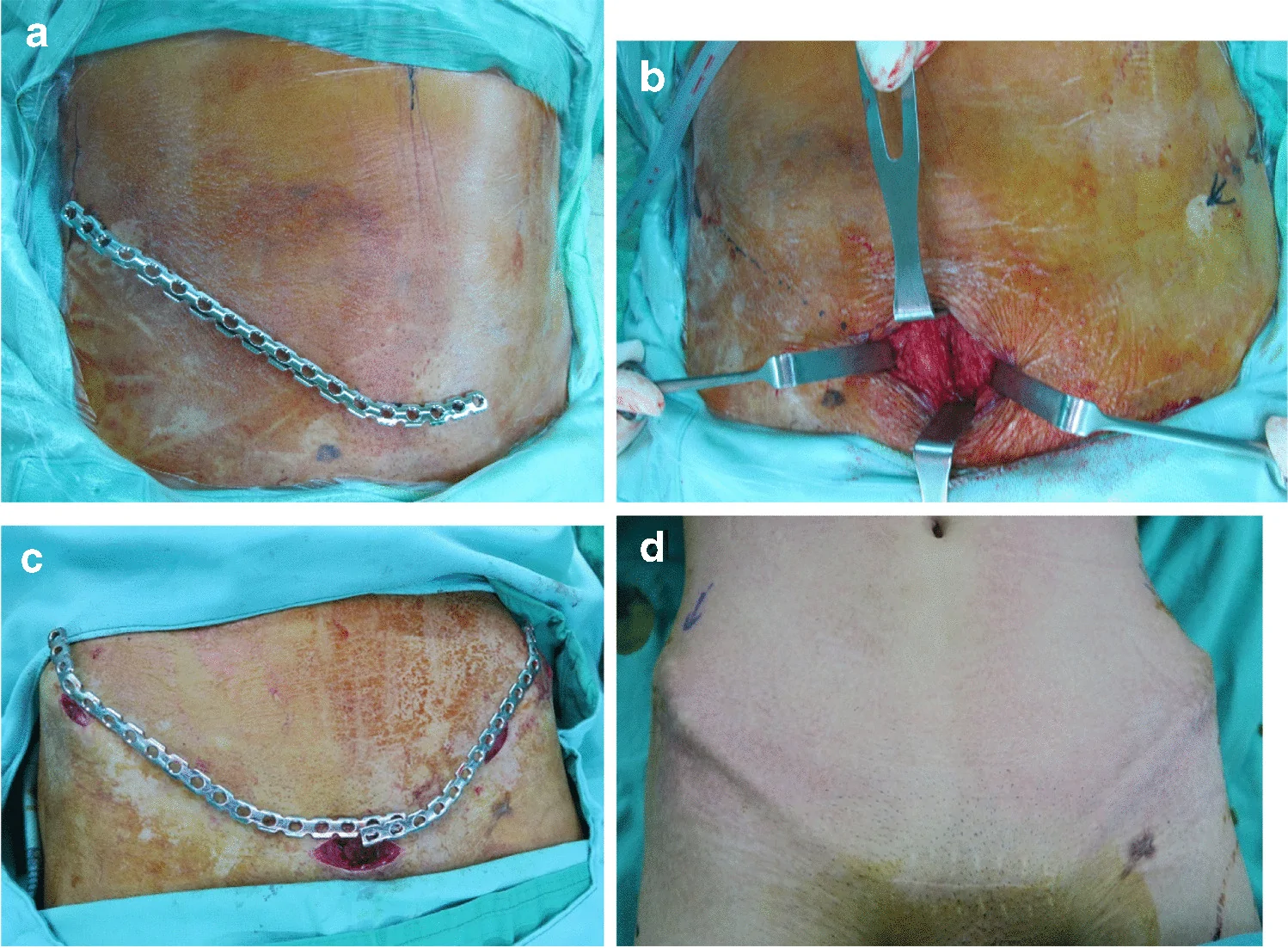
**a** Pre-bending of the plate to fit onto the iliac crest and contralateral pubic tubercle. **b** Skin incision and wound deepened superficial to abdominal fascia. **c** Subcutaneous tunnel created between iliac crest and pubic symphysis. **d** Postoperative image after 6 months of plate implantation

All patients underwent general anaesthesia. Patients were placed in the supine position or, when Kocher–Langenbeck (K–L) approach was involved, in the lateral position. The SAPB technique, originally developed by Cole et al. [7, 8], was used for unstable pelvic ring injuries. SAPB was also termed as the pelvic bridge technique, anterior pelvic internal fixation, percutaneous anterior pelvic bridge, by different studies [7, 9, 10]. A small prebent reconstruction plate (14–18 holes) was fitted onto the iliac crest and contralateral pubic tubercle, superficial to the abdominal fascia. Fixation to ipsilateral tubercle is also feasible, yet fixation to the intact contralateral tubercle can be mechanically advantageous [7, 8, 11]. Bilateral application of the plates was performed if both sides of the acetabular or pelvic lesion were present. Incisions over the anterior portion of the iliac crest and a transverse incision 2 cm above the pubic symphysis were made with a subcutaneous dissection along the inguinal ligament. The plate was inserted subcutaneously anterior to the neurovasculature below and securely fixed with screws at both ends (Fig. 2). For male patients, the spermatic cord was lifted with the plate inserted under the cord, preventing injury to any structure, including the genital branch of the genitofemoral nerve.

### Post-operative management

In addition to daily wound checks, patients were encouraged to perform non-weight-bearing exercises with tolerable pain. Partial weight-bearing was allowed and progressed gradually, depending on each patient’s associated injuries. All patients were followed up at two weeks, one month, three months, and six months post-operatively and annually thereafter.

### Outcome evaluation

Radiographic analysis focused on determining if fracture union had occurred and if the reduction obtained initially was maintained during the healing period. Union of pelvic fractures was determined by the presence of bridging callus, seen at the fracture site on follow-up radiographs. All radiographs were viewed on an electronic picture archiving and communication system (PACS) by an independent examiner and the orthopaedic research fellow, and all measurements were made using PACS-related software. Non-union, malunion, or other complications were also noted. Union beyond 12 weeks was defined as “delayed union” and beyond 24 weeks as “non-union.” Fracture reduction was evaluated by measuring residual displacements on the three post-operative radiographs (one anteroposterior and two 45° oblique Judet views) according to criteria developed by Matta [10]. On the basis of these criteria, postoperative reduction for displaced fracture was graded as anatomical (0–1 mm of displacement), imperfect (2–3 mm of displacement), or poor (> 3-mm displacement). The final follow-up radiographs were graded according to the Matta scoring system [12]. A grading of excellent was given to a normal-appearing hip joint, good to mild for minimal sclerosis and joint narrowing, fair to intermediate for moderate sclerosis and joint narrowing (< 50%), and poor for greater changes. At the final follow-up, a modified version of the clinical grading system developed by d’Aubigné and Postel was used to grade functional outcomes [13]. Avascular necrosis (AVN) of the femoral head was classified according to Ficat and Arlet [14]. Non-displaced fracture was graded by d’Aubigné and Postel scores since no reduction was performed.

## Results

### Demographic data

A total of 21 acetabular fractures with complete data were included and reviewed during the study period. Among these patients, ten were male and eleven were female. Their ages at the time of surgery ranged between 17 and 82 years (mean, 44 years). Eight of the cases involved fracture in the left acetabulum, while the other 13 cases involved the right acetabulum. Seventeen of the cases were displaced fracture (more than 2 mm of displacement), and four of the cases were non-displaced fracture. Regarding the mechanism of injury, 18 cases involved road-traffic accidents (scooter-to-car), and 3 cases involved falling from a height. Besides pelvic fracture, associated injuries were recorded. After initial evaluation and management, pre-operative X-rays of all patients were taken, and CT scan with pelvic 3D reconstruction was performed. All fractures were classified according to AO/OTA criteria (Table 1). The average wait time before operation was 2.1 days. Surgery was performed via anterior pelvic internal fixation (supine position), with 15 cases involving the K–L approach (lateral position) for associated posterior wing fracture. The median operative time was 230 minutes (range 105–830 min), and median blood loss was 100 mL (range 50–1700 mL; Table 1). Because only one patient in our series (no. 11) underwent independent SAPB insertion and other patients had associated injuries that required multiple surgical sites, the average operative time and blood loss were exaggerated. SAPB insertion alone required 60 to 90 minutes of operative time, with minimal blood loss. Most of the patients were mobilized without weight-bearing two days after surgery. Weight-bearing was progressively increased in the following six weeks with full weight-bearing permitted after union of the fractures.

**Table 1:** Clinical and radiological workup of patients with acetabular fractures

No	Sex/age (years)	AO/OTA classification	Postoperative reduction	Associated injuries	BMI	Complications	Removal of implant	Final radiological outcome	Final d’Aubigné and Postel scores
1	Male, 26	62B3.3	Anatomical	Tibial I/L	26	-	-	Excellent	18, excellent
2	Male, 82	62C2e	Anatomical	PR I/L	18	Transient LFCN	-	Good Traumatic OA	15, good
3	Female, 37	62C3.1e	Imperfect	-	26	Loss of reduction	-	Good	16, good
4	Female, 60	62A3.3 61C1.3	Anatomical	Sacrum C/L	27	Morel–Lavallee lesion Transient LFCN	12 months	I/L excellent C/L poor	13, fair
5	Male, 35	62B2.2	Anatomical	Knee avulsion I/L	25	Transient LFCN	7 months	Excellent	17, excellent
6	Female, 48	62A3.1 61C1.2	Anatomical	Ilium, FN, I/L T11 chance	23	-	6 months	Excellent	16, good
7	Male, 52	62C3	Anatomical	Toe amputation, ilium, dislocated hip I/L	27	AVN (THR)	3 months	Excellent	13, fair
8	Male, 22	62C3b	Anatomical	Ilium, PR I/L	28	Transient LFCN	-	Excellent	18, excellent
9	Male, 28	62C1 right 61C3.2a + d left	Anatomical	Ilium I/L, PR I/L, PR C/L, FS B/L	29	-	6 months	Good	16, good
10	Female, 54	62A3.1	Anatomical	Sacrum I/L, Toe	23	-	15 months	Good	18, excellent
11	Male, 69	62B2.2	Anatomical	-	18	-	-	Good	18, excellent
12	Female, 65	62A3.3, 61B2.1a	Anatomical	PR, Sacrum I/L	23	Transient LFCN	20 months	Good	15, good
13	Male, 68	62B2.2	Anatomical	PR C/L	31	Transient LFCN	-	Good Poor C/L	15, good
14	Female, 56	62B3.3	Anatomical	Ribs C/L FN, Tibial I/L	25	-	-	Good	18, excellent
15	Female, 53	62A3.3	Anatomical	L1, Sacrum I/L	28	-	12 months	Good	17, excellent
16	Male, 64	62A3.1	Anatomical	Sacrum I/L PR C/L	25	-	-	Good	18, excellent
17	Female, 24	62A3.3 61C3.1	Anatomical	Sacrum B/L PR C/L	27	-	-	Excellent	18, excellent
18	Female, 21	62A3.1, 61C1.3	Non-displaced	Sacrum I/L	19	-	7 months	Non-displaced	18, excellent
19	Male, 17	62A3.3, 61B1.2	Non-displaced	FS I/L	18	-	18 months	Non-displaced	18, excellent
20	Female, 19	62A3.1 61C3.1 C/L	Non-displaced	PR, Sacrum C/L	17	-	12 months	Non-displaced	18, excellent
21	Female, 30	62A3.1 61C3.1	Non-displaced	Clavicle, PR C/L	21	Transient LFCN	6 months	Non-displaced	15, good

*BMI* body mass index, *FN* femoral neck, *FS* femoral shaft, *PR* pubic ramus, *IW* iliac wing, *B/L* bilateral, *C/L* contralateral, *I/L* ipsilateral, *LFCN* lateral femoral cutaneous nerve, *AVN* avascular necrosis, *THR* total hip replacement

### Radiographic outcome

At the final follow-up, the radiographic outcome of displaced fracture according to the Matta system revealed excellent radiological outcomes in seventeen displaced fractures with seven excellent outcomes and nine good outcomes. One case of non-union pubic ramus fracture from a traumatic fracture in the contralateral pubic ramus was observed in our series. One patient had anterior column screw loosening with limitation of hip movement one week after the initial operation, and a secondary revision operation was performed.

### Functional outcome scores

At the final follow-up, the functional outcome according to the d’Aubigné and Postel grading system revealed excellent results in twelve hips, good results in seven, and fair results in two (Table 2).

**Table 2:** Radiological and functional outcomes of patients at final follow-up

Fracture reduction	Radiological outcome				Functional outcome				AVN
	Excellent	Good	Fair	Poor	Excellent	Good	Fair	Poor	
Anatomical (*n*)	7	9	-	-	9	5	2	-	1
Imperfect (*n*)	-	1	-	-	-	1	-	-	-
Non-displaced (*n*)	4	-	-	-	3	1	-	-	-

*AVN* avascular necrosis

### Complications

Regarding surgical and fracture-healing complications during follow-up, no surgical site, deep infection, or other wound-healing complications were observed. Six patients sustained transient LFCN palsy and presented with numbness or paraesthesia that resolved within 3 months. One patient had anterior column screw loosening with limitation of hip movement one week after the initial operation, and a secondary revision operation was performed. One patient developed AVN of the left femoral head after three months, and total hip replacement along with removal of the previous implant was performed.

### Removal of implants

Implants were typically removed after 6 months, after fracture healing had been demonstrated on X-ray. Among implants which were removed, the mean time to removal was 10.3 months. One patient had the implant removed after 3.5 months because of intolerable discomfort. Nine patients did not have their implants removed after one year of follow-up because of the current (COVID-19) pandemic or tolerable discomfort with little limitation of movement.

## Discussion

Current common operative methods for displaced anterior wall or column fractures are the ilioinguinal and modified Stoppa approaches. The ilioinguinal approach was developed by Letournel and Judet [15] (who standardized the classification of acetabular fractures) and was adopted as the standard method for ORIF in acetabular fractures. Rives et al. [16] and Stoppa et al. [17] developed an intra-abdominal surgical approach for the repair of groin hernias, the modification of which was used by Cole and Bolhofner as a surgical approach for ORIF in acetabular fractures (i.e., modified Rives–Stoppa approach) [6]. Recently, Cole et al. [7] described a novel approach in which subcutaneous plates are inserted through minimal incisions over the ilium, with fixation to the contralateral ilium or pubic symphysis. This technique was later termed anterior pelvic internal fixation (APIF) and has been used to treat anterior pelvic ring disruptions. Both the ilioinguinal and modified Stoppa approaches require intrapelvic access, which has the potential of damaging several structures [6, 15]. In the ilioinguinal approach, the medial lymphatics and external iliac veins are suspended, and any damage to the external iliac veins could cause major bleeding.

Common treatment for non-displaced acetabular fractures and fractures that do not involve the weight-bearing acetabular dome is conservative [18]. However, patients often suffer from chronic pain with difficult early mobilization, prolonged recumbency, and subsequently request for surgical intervention [19]. In order to prevent extensive exposure from traditional surgical treatment, percutaneous lag screws fixation is often considered for this type of patient [2]. Nonetheless, fluoroscopy-guided percutaneous screw fixation remains a technically demanding procedure with possible neurovascular complications while CT-guided fixation required relatively uncommon equipment for intra-operative 3D imaging [20].

The subcutaneous anterior pelvic bridge is a minimally invasive treatment for selective acetabular fracture that is performed without peeling the iliopsoas and femoral vessels and nerves [7, 8, 11]. Quick pain relief, rapid post-operative mobilization with minimal soft tissue damage, and relatively short learning curve are the main advantages. In our study, 16 of our 17 displaced fracture cases achieved anatomical reduction after initial surgery. Blood loss in the APIF approach was minimal when SAPB was the sole implantation; other blood loss was due to associated injuries and the K–L approach. Posterior pelvic ring stability is required for this technique; thus, the K–L approach for posterior column fracture is also often performed jointly. While performing the K–L approach, we implemented the Lien traction scaffold from our previous study for constant and precise mechanical traction [21]. The operative time of our SAPB implantation was between 60 and 90 min, with the majority of the operative time spent on the K–L approach or associated injuries. No wound infection, recurrent dislocation, or pulmonary embolism was noted in our follow-up. Anatomical reduction of was achieved in 94.1% patients, which is comparable with the rates (80–90%) reported in the literature [5, 6].

The selected types of acetabular fractures were confirmed as anterior wall or column fractures, transverse fractures below the illiopubic eminence, or both column fractures. In line with the studies of Konrath et al. [22, 23], high-anterior column fracture showed increased peak contact pressures with both step and gap malreductions, and low anterior wall fractures corresponding to a 45° roof arc measurement had no effect on peak pressure or contact area within the superior acetabulum. We concluded these studies to select suitable cases for SAPB, as weight-bearing force was not primary loaded onto the anterior region of acetabulum but instead onto the superior region which was articulated directly with the femoral head [24]. Therefore, SAPB should provide enough stability for low anterior wall or column fractures, which is also demonstrated in our study. We suggest treating displaced high-anterior column fracture with traditional ilioinguinal or modified Stoppa approach as SAPB would be insufficient for reduction. However, for both column fractures, high-anterior column fracture could be treated by SAPB with K–L approach, as posterior K–L approach would achieve pelvic wing reduction and stability. Our study also suggests that anatomical reduction of the weight-bearing region would produce an excellent functional outcome.

**Fig. 3:**
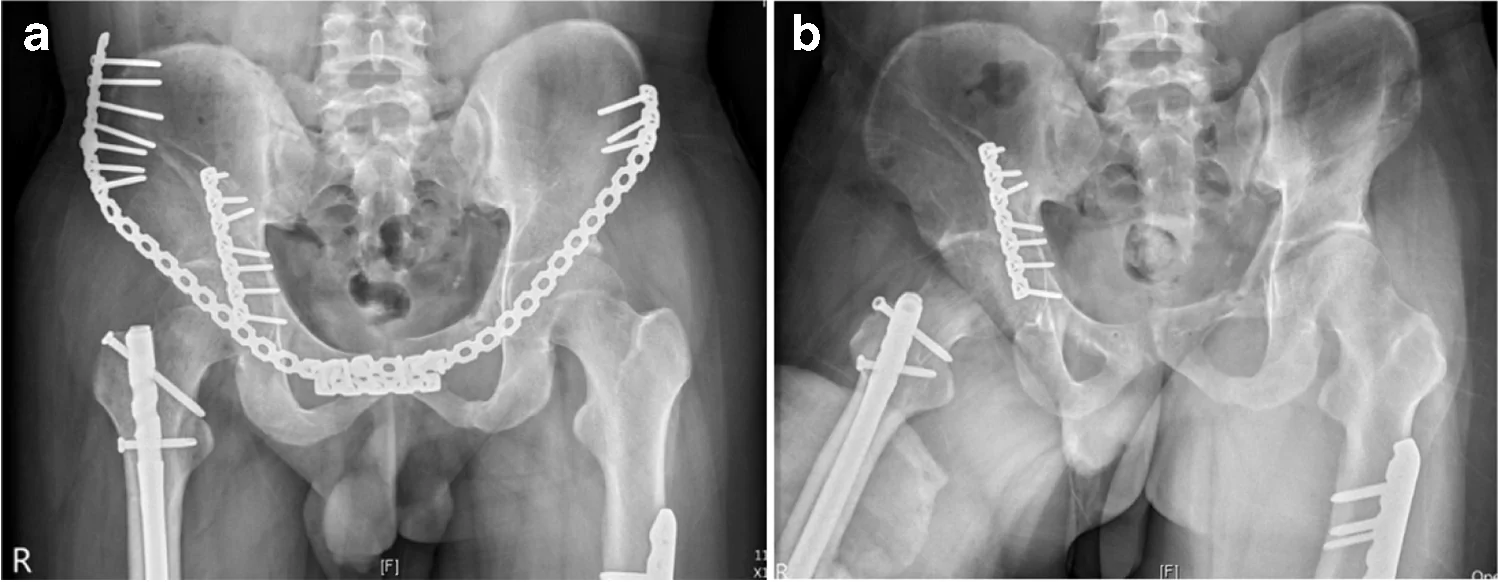
**a** Post-operative radiographs of a 28-year-old man (no. 9) showing right iliac wing fracture, right acetabular both column fracture, left superior and inferior pubic rami fracture, pubic symphysis diastasis, and bilateral femoral open fracture showing anatomical reduction. **b** AP radiographs after removal of SAPB showing excellent radiological outcome with excellent functional outcome

**Fig. 4:**
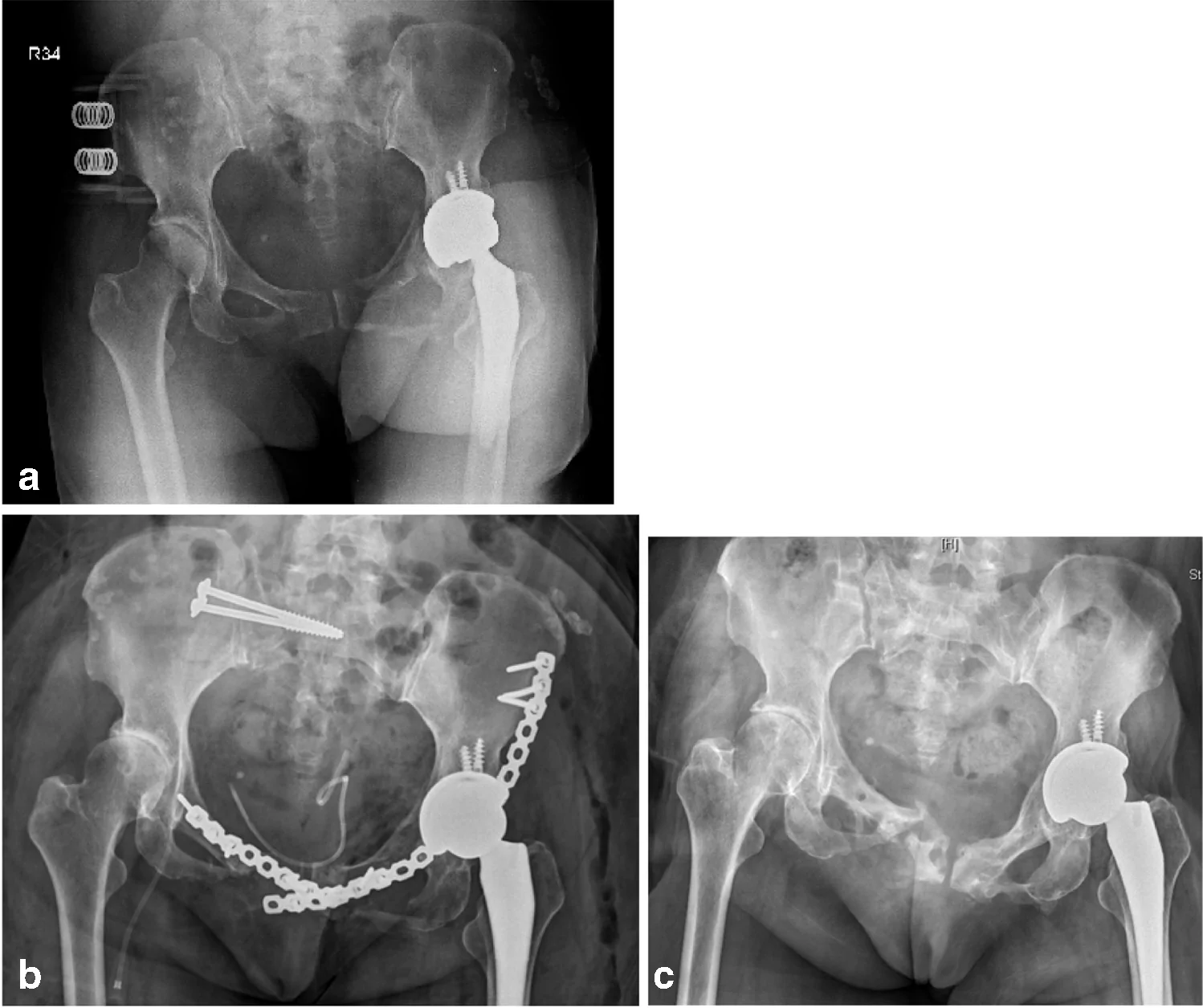
**a** Pre-operative AP radiographs of a 60-year-old woman with history of left THR (no. 4), showing left low anterior column fracture with right saddle type pubis fracture and sacral zone II fracture. **b** Post-operative AP radiograph showing anatomical reduction. **c** AP radiographs after removal of implants showing poor radiological outcome on right side pubic ramus but excellent radiological outcome on left hip

**Fig. 5:**
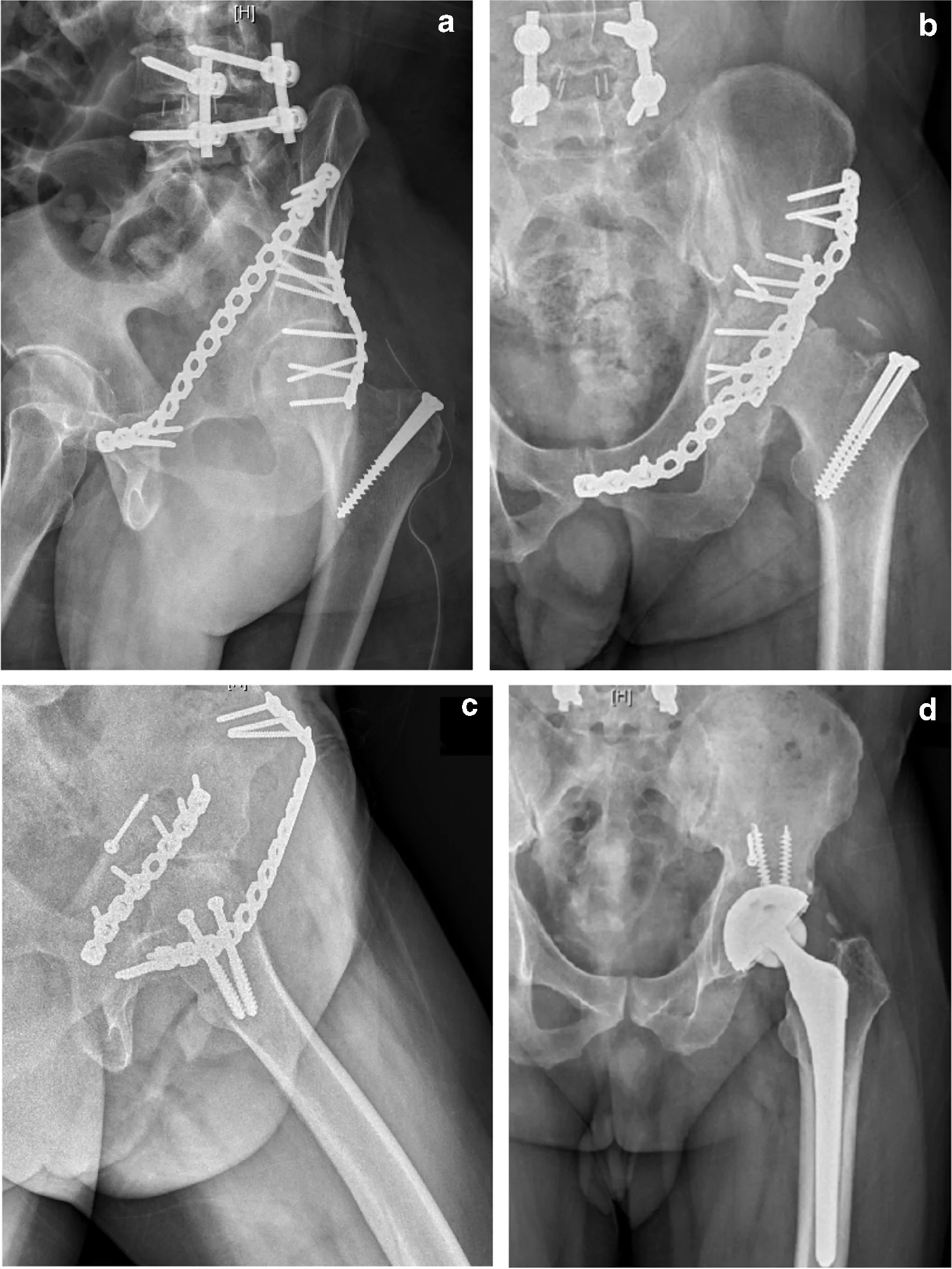
**a** Post-operative Judet view radiographs of a 52-year-old man (no. 7) showing reduction of left ilium wing, left acetabular (two-column) central complex fracture with dislocated left hip joint and split femoral head fracture. **b** Pelvis AP and Judet view radiographs after 3 months of surgery showing signs of avascular necrosis and subcapital fracture. **e** AP radiograph after total hip replacement and removal of SAPB showing excellent radiological outcome but fair functional outcome

Although, in the study of He et al. [25], SAPB produced less biomechanical stability of the pelvic ring compared with laparoscopic-assisted plate and transramus intraosseous screw, we achieved mostly satisfactory functional outcomes and adequate stability. In our series, we observed that four patients aged <30 years had fast recovery and excellent d’Aubigné and Postel score of 18 3 months after operation; one of these patients (no. 1) could not remove the implant because of the (COVID-19) pandemic, but the implant did not prevent him from any athletic activity. However, a palpable plate in the subcutaneous layer of the patient, causing discomfort such as mild limitation of hip and groin pain, was the main disadvantage. Other complaints included thigh numbness and soreness, especially with prolonged posture or lateral lying on the side of implant. Most patients’ complaints resolved after three months of follow-up or after removal of the implant. For patients that had implant-related symptoms of discomfort or neurological signs, the implants were typically removed after fracture healing, which required about 6 months based on our experience. Malunion, implant failure, and activity levels of patients were other considered parameters. Patients whose complaints persisted were mostly patients with multiple trauma (Figs. 1d and 3) or spine injury. These patients would often present with lower back pain or sciatica pain radiating down the leg. Further imaging or electromyography would eventually be required for these patients. In our study, one patient (Fig. 4) developed Morel–Lavallee lesion and was managed with fasciotomy and fasciectomy, along with antibiotics and daily redressing. This patient also had a poor radiologic outcome on the contralateral side of the pubic ramus (by ORIF approach), accompanied by hip tightness and movement pain, but excellent outcome on the anterior column fracture (by SAPB approach). We speculate inadequate fixation by reconstruction plate on the small tuberosity fragment led to loss of reduction and non-union of the pubic ramus six months after initial surgery; the ORIF approach could also have caused additional soft tissue damage and delayed healing. A study by Huang et al. [10] provided a novel approach to fixing K-wires on the pubic symphysis, which could improve the quality of reduction and provide more stability to this type of fracture. One patient (Fig. 5) had a dislocated left hip joint and femoral head split fracture and was managed with the K–L approach along with trochanteric osteotomy fixed with two 4.5-mm cancellous screws. Three months later, the patient presented with persistent hip pain, and AVN of the left femoral head with subcapital fracture was diagnosed. Total hip replacement along with removal of the previous implant was then performed. A study by Barquet et al. [26] suggested that the disruption of the extra-osseous arterial blood supply to the femoral head during the operative procedure seems to be the most probable developmental pathway of AVN, although high-energy injury with fracture displacement could also be a risk factor [26]. Although the rates of this complication are low [27], a careful postoperative follow-up for monitoring signs of AVN is essential.

Our study is somewhat limited by its retrospective nature and relatively small population size, although these limitations are common to most series on anterior acetabular wall fractures [4]. Traumatic osteoarthritis was not evaluated in this research because it would require additional long-term follow-up. This was a single-institution study, with all patients operated on by the same surgical team. Despite the small population size, we established that the approach was simple and had satisfactory outcomes and few complications. Overall, these shortcomings could be resolved by future prospective cohort study and randomized investigations. A longer-term follow-up will demonstrate the true success of this implant, and we continue to keep this cohort of patients under review.

## Conclusions

The innovative subcutaneous anterior pelvic bridge for the treatment of selective acetabular fracture is proven to be a feasible method with promising outcomes. This approach, compared with the more invasive traditional ilioinguinal or modified Stoppa approach, is a minimally invasive technique and strengthens the stability of fixation, with less blood loss and fewer intra-operative/post-operative complications. Our clinical experience supports recommending this innovative method to orthopaedic surgeons.
